# Supplementary material for: Global characterization of copy number variants in epilepsy patients from whole genome sequencing
Source: PLoS Genet. 2018 Apr 12;14(4):e1007285. doi: 10.1371/journal.pgen.1007285 (PMC5978987; doi:10.1371/journal.pgen.1007285)
Supplement: S1 Text — (PDF) [file pgen.1007285.s001.pdf]

Supplementary Material for "Global characterization of copy number variants in epilepsy patients from whole genome sequencing"

Jean Monlong[1,2,¶], Simon L. Girard[1,3,4,¶], Caroline Meloche[4], Maxime Cadieux-Dion[4,5], Danielle M. Andrade[6], Ron G. Lafreniere[4], Micheline Gravel[4], Dan Spiegelman[7], Alexandre Dionne-Laporte[7], Cyrus Boelman[8], Fadi F. Hamdan[9], Jacques L. Michaud[9], Guy Rouleau[7], Berge A. Minassian[10], Guillaume Bourque[1,2,11,*], and Patrick Cossette[4,*]

[1]Department of Human Genetics, McGill University, Montréal, H3A 1B1, Canada

[2]Canadian Center for Computational Genomics, Montréal, H3A 1A4, Canada

[3]Département des sciences fondamentales, Université du Québec à Chicoutimi, Chicoutimi, G7H 2B1, Canada

[4]Centre de Recherche du Centre Hospitalier de l'Université de Montréal, Montréal, H2X 0A9, Canada.

[5]Center for Pediatric Genomic Medicine, Children's Mercy Hospital, Kansas City, MO, USA

[6]Epilepsy Genetics Program, Division of Neurology, Toronto Western Hospital, University of Toronto, Toronto, Canada.

[7]Montreal Neurological Institute, McGill University, Montréal, H3A 2B4, Canada.

[8]Division of Neurology, BC Children's Hospital, Vancouver, V6H 3N1, Canada

[9]CHU Sainte-Justine Research Center, Montréal, H3T 1C5, Canada.

[10]Division of Neurology, The Hospital for Sick Children, Toronto, M5G 1X8, Canada.

[11]McGill University and Génome Québec Innovation Center, Montréal, H3A 1A4, Canada

[¶]These authors contributed equally to this work

*Correspondence: guil.bourque@mcgill.ca (GB), patrick.cossette@umontreal.ca (PC)

Contents

1 Epilepsy patients and sequencing **2**

2 Testing for technical bias in WGS **4**

3 PopSV **5**

4 Validation and benchmark of PopSV **9**

5 CNV detection in the CENet cohorts **12**

6 CNV enrichment in exonic region and around epilepsy genes **12**

1 Epilepsy patients and sequencing

Ethics and patients recruitment CENet is a Genome Canada and Genome Québec funded initiative that aims to bring personalized medicine in the treatment of epilepsy. Patients were recruited through two main recruitment sites at the Centre Hospitalier Universitaire de Montréal (CHUM) and the Sick Kids Hospital in Toronto. This study was approved by the Research Ethics Board at the Sick Kids Hospital (REB number `1000033784`) and the ethics committee at the Centre Hospitalier Universitaire de Montréal (project number `2003-1394,ND02.058-BSP(CA)`). Before their inclusion in this study, patients had to give written informed consents. The main cohort of this study was constituted of 198 unrelated patients with various types of epilepsy; 85 males and 113 females. The mean age at onset of the disease for our cohort was 9.2 (±6.7). S1 Table presents a detailed description of the clinical features for the various individuals recruited in this study. DNA was extracted from blood DNA exclusively. 301 unrelated healthy parents of other probands from CENet were also included in this study and used as controls.

Libraries preparation and sequencing gDNA was cleaned up using ZR-96 DNA Clean & Concentrator^{TM}-5 Kit (Zymo) prior to being quantified using the Quant-iT^{TM} PicoGreen® dsDNA Assay Kit (Life Technologies) and its integrity assessed on agarose gels. Libraries were generated using the TruSeq DNA PCR-Free Library Preparation Kit (Illumina) according to the manufacturer's recommendations. Libraries were quantified using the Quant-iT^{TM} PicoGreen® dsDNA Assay Kit (Life Technologies) and the Kapa Illumina GA with Revised Primers-SYBR Fast Universal kit (Kapa Biosystems). Average size fragment was determined using a LabChip GX (PerkinElmer) instrument.

The libraries were first denatured in 0.05N NaOH and then were diluted to 8pM using HT1 buffer. The clustering was done on a Illumina cBot and the flowcell was run on a HiSeq 2500 for 2x125 cycles (paired-end mode) using v4 chemistry and following the manufacturer's instructions. A phiX library was used as a control and mixed with libraries at 1% level. The Illumina control software was HCS 2.2.58, the real-time analysis program was RTA v. 1.18.64. Program bcl2fastq v1.8.4 was then used to demultiplex samples and generate fastq reads. The average coverage was 37.6x $\pm$ 5.6x. The filtered reads were aligned to reference Homo_sapiens assembly b37. Each readset was aligned using BWA[6] which creates a Binary Alignment Map file (.bam). Then, all readset BAM files from the same sample are merged into a single global BAM file using Picard. Insertion and deletion realignment was performed on regions where multiple base mismatches were preferred over INDELs by the aligner since it appears to be less costly for the algorithm. Such regions were found to introduce false positive variant calls which may be filtered out by realigning those regions properly. Once local regions were realigned, the read mate coordinates of the aligned reads needed to be recalculated since the reads are realigned at positions that differ from their original alignment. Fixing the read mate positions is performed using Picard. Aligned reads were marked as duplicates if they have the same 5' alignment positions (for both mates in the case of paired-end reads). All but the best pair (based on alignment score) were marked as a duplicate in the `.bam` file. Duplicates reads were excluded in the subsequent analysis. Marking duplicates was performed using Picard.

2 Testing for technical bias in WGS

To investigate the bias in read depth (RD), we first fragmented the genome in non-overlapping bins of 5 Kbp. The number of properly mapped reads was used as RD measure, defined as read pairs with correct orientation and insert size, and a mapping quality of 30 (Phred score) or more. In each sample, GC bias was corrected by fitting a Loess model between the bin's RD and the bin's GC content. Using this model, the correction factor for each bin was estimated from its GC content. Bins with extreme coverage were identified when deviating from the median coverage by more than 3 standard deviation. After these conventional intra-sample corrections, RD across the different samples were combined and quantile normalized. At that point the different samples had the same global RD distribution and no bins with extreme coverage or GC bias. Two control RD datasets were constructed to represent our expectation when no bias is present. One was derived from the original RD by shuffling the bins' RD in each sample. In the second, RD was simulated from a Normal distribution with mean and variance fitted to the real distribution. Simulation or shuffling ensures that no region-specific or sample-specific bias remains. To investigate region-specific bias, we computed the mean and standard deviation of the RD in each bin across the different samples. The same was performed in the control datasets. If there is no bias, the distribution of these estimators should be similar in the original, shuffled and simulated RD. Next, to investigate experiment-specific bias, we retrieved which sample had the highest coverage in each bin. Then we computed, for each sample, the proportion of the genome where it had the highest coverage. If no bias was present, e.g. in the shuffled and simulated datasets, each sample should have the highest coverage in 100/N % of the genome (with N the number of samples). If an experiment was more affected by technical bias, it would be more often extreme. The same analysis was performed monitoring the lowest coverage.

The same analysis was ran after correcting the coverage in the Twin dataset using the QDNAseq pipeline[1]. The reads were counted in 5 Kbp bins using the function `binReadCounts`. GC bias and mappability were corrected using the following functions (with default parameters): `applyFilters`, `estimateCorrection`, `correctBins`, `normalizeBins`, `smoothOutlierBins`.

3 PopSV

Binning and coverage measure The genome is fragmented in non-overlapping consecutive bins of fixed size (5 Kbp). In each bin and each sample the number of reads that overlap the bin and are properly mapped are counted to get a measure of coverage. Read pairs with correct orientation and insert size, and a mapping quality of 30 (Phred score) or more are considered properly mapped. The bin counts were then corrected for GC bias. In each sample, a LOESS model was fitted between the bin's count and bin's GC content. A normalization factor was then defined for each bin from its GC content.

Constructing the set of reference samples In the epilepsy study and the Twins dataset we used all the samples as reference. In the renal cancer dataset we used the normal samples as reference. For each dataset, a Principal Component Analysis (PCA) was performed across samples on the counts normalized globally (median/variance adjusted). The resulting first two principal components are used to verify the homogeneity of the reference samples. In the presence of extreme outliers or clear sub-groups, a more cautious analysis would be recommended. For example, outliers can remain in the set of reference samples but flagged as they might potentially harbor more false calls later. Independent analysis in each of the identified sub-group is also a solution, especially when the same samples are to be used as reference. Although our three datasets showed different levels of homogeneity, we did not need to exclude samples or split the analysis. The effect of weak outlier samples was either corrected by the normalization step or integrated in the population-view. Moreover, the principal components were used to select one control sample from the final set of reference samples. This sample is used in the normalization step as a baseline to normalize other samples against. We picked the sample closest to the centroid of the reference samples in the Principal Component space.

Normalization Although uniformity of the coverage across the genome is not required for our approach, RD values must be comparable across samples. When a particular region of the genome is tested, sample specific variation of technical origin must be minimized. This is done through a normalization step. Naive global normalization approaches like the Trimmed-Mean M(TMM) or

quantile normalization have been first implemented and tested. The TMM normalization robustly aligns the mean RD value in the samples. Quantile normalization forces the RD distribution to be exactly the same in each sample. After witnessing the presence of uncharacterized sample-specific variation, we implemented a more suited normalization. Targeted normalization uses information across the set of reference samples to identify similar bins across the genome and normalize their counts separately (S19 Fig). For each bin, the top 1000 bins with similar coverage patterns across the reference samples are used to normalize the coverage of the bin. TMM normalization is used on these top 1000 bins to derive the correct normalization factor for the bin to normalize. Similarity between two bins is measured using Pearson correlation between the counts across the reference samples. Hence, the top 1000 bins are most similar in term of relative coverage across the samples to the coverage in the bin to normalize. If some bias is present in some samples, the top 1000 bins should also harbor this bias. Hence, other regions with similar bias patterns are used to correct for it. In this targeted approach, each genomic region is normalized independently. The 1000 supporting bins are saved and used to normalized new samples (e.g. case sample). Although computationally expensive, it ensures that all bins are normalized with the same effort. In contrast, global normalization or even PCA-based approaches corrects for the most common or spread bias, but a subset of regions with specific bias might not be corrected. In order to compare the performance of the different normalization approaches we computed a set of quality metrics. The normalized RD will need to be suited for testing abnormal pattern across samples: under the null hypothesis, i.e. for normal bins, the RD should be relatively normally distributed and the samples rank should vary randomly from one bin to the other. The first metric is the proportion of bins with non-normal RD across the samples. Shapiro test was performed on each bin and a P-value lower than 0.01 defined non-normal RD. Then, the randomness of the sample ranks was tested by comparing the RD of each sample a region with the median across all samples. In regions of 100 consecutive bins, we counted how many times the RD in a sample was higher than the median across sample. If the ranks are random, this value should be around 0.5. The probability under the Binomial distribution is computed for each sample and corrected for multiple testing using Bonferroni correction. If any sample has an adjusted P-value lower than 0.05, we consider that the region has non-random ranks. The resulting QC metric is simply the proportion of regions with non-random sample ranks. This

QC is specifically testing how much sample-specific bias remains. The remaining QC metrics look at the Z-score distribution in each sample. The proportion of non-normal Z-scores is computed by comparing the density curves of the Z-scores and simulated Normal Z-scores. We compute the proportion of the area under the density curve that does not overlap the Normal density curve. This estimate of the proportion of non-normal Z-scores is computed in each sample. The final metrics are the average and maximum across the samples.

Abnormal RD test and Z-score computation The test is based on Z-scores computed for each bin, corrected afterward for multiple testing. The Z-score represents how different the read count in the tested sample is from the reference samples. It is simply: $z = \frac{BC_t^b - mean(BC_{ref}^b)}{sd(BC_{ref}^b)}$ where BC_t^b is the bin count, i.e. the number of reads, in bin b and sample t. Inevitably some samples are hosting common CNVs. We observed that just a couple of samples hosting a CNVs could be enough to bias the standard deviation used in the score computation and mask these CNVs in the coming tests. In many cases the RD signal was clearly showing several groups of samples with proportional read counts. To improve the Z-score computation in those regions, a simple approach was used: the samples were stringently clustered using their RD and the group with higher number of samples was chosen as reference and used to compute the mean and standard deviation for the Z-score computation. In practice, this clustering affects only bins with clear clusters but would remove just a few or no samples in most situations. Furthermore, a median-based estimator was used for the standard deviation as it is less sensitive to outlier removal. A trimmed mean was also preferred over normal mean for its robustness to outliers.

Significance and multiple testing correction The Z-scores for all the bins of a sample are pooled and significance is estimated. Under the null hypothesis of normally distributed read counts, the Z-scores should also follow a normal distribution. For multiple testing correction, the Z-score empirical distribution is used to fit a normal and estimate the P-value and Q-value of each test. This step is performed using fdrtool R package. By default, the null distribution fitting for P-value computation assumes that only a low proportion of bins violates the null hypothesis. In aberrant genomes, e.g. in tumor samples, it is often an unrealistic assumption. We devised a new strategy

to set the proportion of the empirical distribution, later used to estimate the null distribution variance. Here the null Z-score distribution is assumed to be centered on 0 and its variance is estimated by trimming the tails of the empirical distribution. To find a correct trimming factor, an iterative approach started from a low trimming factor and increased its value until reaching a plateau for the variance estimator. Indeed, once the plateau is reached, additional trimming does not change the estimated variance because there is no more abnormal Z-scores, only the central part of the null distribution. Samples with an important proportion of abnormal genome, e.g. tumor samples, showed more appropriate fit. Of note, the P-values for positive Z-scores (duplication) and negative Z-scores (deletion) are estimated separately. Thus, imbalance in the deletion to duplication ratio, or large aberration that lead to asymmetrical Z-score distribution does not affect the P-value estimation. Multiple testing correction is performed after pooling all the P-values.

Segmentation, copy number estimation and other metrics Following the significance estimation, consecutive bins with abnormal coverage are merged into a call. Consecutive or nearby abnormal bins (e.g. one bin size apart) are merged into one variant if in the same direction (deletion or duplication). In PopSV's R package, the P-values can also be segmented using circular binary segmentation[7].

In addition to the Z-score, P-value, Q-value and number of bins of each call, PopSV retrieves the average coverage in the reference samples and the fold change in the sample tested. The copy number is estimated by dividing the coverage in a region by the average coverage across the reference samples, multiplied by 2 (as diploidy is expected). In our bin setting, the estimation is correct if the bin spans completely the variant. For this reason we trust the copy number estimate for calls spanning 3 or more consecutive bins, as it is computed using the middle bin(s) which completely span the variant. In other cases we expect the copy number estimate to be under-estimated. All this additional information can be used to order or retrieve high confidence calls. For examples, several consecutive bins or a copy number estimate around an integer value increases our confidence in a call. In our benchmark, we used the entire set of calls.

4 Validation and benchmark of PopSV

We compared PopSV to FREEC[8], CNVnator[9] and cn.MOPS[10], three popular RD methods that can be applied to WGS datasets to identify CNVs. FREEC segments the RD values of a sample using a LASSO-based algorithm while CNVnator uses a mean-shift technique inspired from image processing. cn.MOPS considers simultaneously several samples and detects copy number variation using a Poisson model and a Bayesian approach. We also ran LUMPY[11] which uses an orthogonal mapping signal: the insert size, orientation and split mapping of paired reads.

FREEC and CNVnator were run on each sample separately, starting from the BAM file. FREEC internally corrects the RD for GC and mappability bias. In order to compare its performance across the entire genome, the minimum telocentromeric distance was set to 0. The remaining parameters were set to default. Of note an additional run with slightly looser parameter ('breakPointThreshold=0.6') was performed to get a larger set of calls used in some parts of the in silico validation analysis to deal with borderline significant calls. CNVnator also corrects internally for GC bias. We used default parameters. For the analysis using higher confidence calls, we used calls with either 'eval1' or 'eval2' lower than 10-5 (instead of the default 0.05). cn.MOPS was run on the same GC-corrected bin counts used for PopSV. All the samples are analyzed jointly. Of note an additional run with slightly looser parameter ('upperThreshold=0.32' and 'lowerThreshold=-0.42') was performed to get a larger set of calls used in some parts of the in silico validation analysis to deal with borderline significant calls. For LUMPY, the discordant reads were extracted from the BAMs using the recommended commands. Split-reads were obtained by running YAHA[12] with default parameters. All the CNVs (deletions and duplications) larger than 300 bp were kept for the upcoming analysis. Calls with 5 or more supporting reads were considered high-confidence.

First, we compared the frequency at which a region is affected by a CNV using the calls from the different methods. In order to investigate how many systematic calls are present in a typical run, we compare the frequency distributions on average per sample. In S4 Fig, the bars represents the average proportion of a sample's calls in each frequency range.

Then, the samples were clustered using the CNV calls. The distance between two samples A and B is defined as : $1 - 2\frac{|VAB|}{(|VA|+|VB|)}$ where VA represents the variants found in sample A,

VAB the variants found in both A and B, and |V| the cumulative size of the variants. Hence, the similarity between two samples is represented by the amount of sequence called in both divided by the average amount of sequence called. This distance is used for hierarchical clustering of the samples. Different linkage criteria ("average", "complete" and "Ward") were used for the exploration. In our dendograms we used the "average" linkage criterion. The same clustering was performed using only calls in regions with extremely low coverage (reference average $<$10 reads).

To assess the performance of each method, we measured the number of CNVs identified in each twin that were also found in the matching twin. In order to avoid missing calls with borderline significance, we used slightly less confident calls for the second twin. We removed calls present in more than 50% of the samples to ensure that systematic errors were not biasing our replication estimates. Hence, a replicated call is most likely true as it is present in a minority of samples but consistently in the twin pair. Even if we removed systematic calls, the most frequent calls in the cohort are more likely to look replicated by chance, compared to rare calls. To normalize for this effect, we use the frequency distribution to compute the number of replicated calls expected by chance. In practice the null concordance for each call is simulated by a Bernoulli distribution of parameter the frequency of the call. This number of replicated calls by chance is subtracted to the original number of replicated calls to give an adjusted measure of sensitivity. Although we do not know the true number of variant, this number of replicated calls is used to compare the different methods. When possible, the low-quality calls were also gradually filtered to explore the effect on the replication metrics. For CNVnator, we used the minimum of the eval1 and eval2 columns, with lower values corresponding to higher quality calls. For LUMPY, the number of supporting reads was used. For PopSV, we filtered calls based on adjusted P-values.

In addition to their replication, we compared which regions were called by several methods. For each of the calls found in less than 50% of the samples, we overlapped the region with calls from other methods in the same sample. If calls from another method overlapped we considered the call shared and saved which methods shared the call. To focus on on high quality calls we considered calls found by at least two methods and computed the proportion of calls from one method found by each of the other methods. This metric captures how much each method recovers high-quality calls from a second method.

Concordance between different bin sizes We compared calls using small bins (500 bp) and calls using larger bins (5 Kbp). In theory, calls from the 5 Kbp analysis should be supported by many 500 bp calls. We also expect large stretches of 500 bp calls to be detected in the 5 Kbp analysis. This comparison is informative as it explores the quality of the calls, the size of detectable events and the resolution for different bin sizes. First we counted how many small bin calls supported any large bin call. These metrics were separated according to the size of the large bin call. Overall, we find that 5 Kbp calls are well supported by 500 bp calls, with only 14% of the 5 Kbp bins not supported by any 500 bp bin (S9 Fig). To investigate large bin calls with no supporting small bin call, we display the average Z-scores in the small bins overlapping large bin calls to test if the lack of support is due to lower confidence or real discordancy between the different runs. If the Z-scores in the small bins deviates from 0 in the correct direction, we conclude that they support the large bin call. Even for these unsupported 5 Kbp calls, we find that the 500 bp bins RD was consistently enriched (or depleted) although not enough to be called with confidence (S9 Fig). This is expected given the higher background noise in the 500 bp analysis that will reduce the power to call these variants. Next, we looked at the proportion of 500 bp calls, grouped by size, that were found in the 5 Kbp calls. More specifically, we grouped them by size to verify that large enough small bin calls are present in the large bin calls. This analysis is used to both test the sensitivity of PopSV with a particular bin size, and its resolution to variants smaller than the bin size. Indeed, this framework allow us to ask questions such as: how much of the variants spanning only half a bin are detected ? We find that the concordance gradually increases until the 500 bp calls reach 5 Kbp in size where the concordance rises to nearly 100% (S9 Fig). This suggests that PopSV is able to detect approximately 75% of the events as large as half its bin size, and almost all events larger than its bin size. As expected, only a small proportion of the small 500 bp calls overlap 5 Kbp calls and they likely corresponds to fragmented larger calls. Considering the trade-off between bin size and noise, this suggests running PopSV with a few bin sizes to better capture variants of different sizes.

5 CNV detection in the CENet cohorts

CNVs were called using PopSV using 5 Kbp bins and all the samples from both the epilepsy and control cohorts as reference. We annotated the frequency of the CNVs using germline CNV calls from the Twin and cancer datasets (internal database) as well as four public CNV databases:

- CNVs from Phase 1 of the 1000 Genomes Project as identified by Genome STRiP[13].
- SV from the 1000 Genomes Project phase 3[14].
- Genome of Netherlands[15].
- CNVs from the Simons Genome Diversity Project[16].

CNVs were annotated with the maximum frequency in the databases. For each CNV to annotate, any overlapping CNV in the CNV databases were considered. This is a stringent criterion that ensures that the entire regions of a rare CNV, for example, is never affected by common CNVs in the databases. Hence, a rare CNV is defined as present in less than 1% of the samples in each of the five CNV databases.

To test for a difference in deletion/duplication ratio among rare CNVs, we compared the numbers of rare deletions and duplications in the epilepsy patients and controls using a χ^2 test. The same test was performed after downsampling the controls to the sample size of the epilepsy cohort.

6 CNV enrichment in exonic region and around epilepsy genes

Enrichment in exons For each cohort, we retrieved the CNV catalog by merging CNV that are recurrent in multiple samples. Hence, the CNV catalog represents all the different CNVs found in each cohort. To control for the population size, we sub-sampled 150 samples in each cohort a hundred times. For each sub-sampling and each cohort, control regions are selected to fit the size distribution of the CNV catalog and the overlap with centromere, telomeres and assembly gaps (details in the next section).

Then, we computed the proportion of CNV and control regions that overlap an exon. The fold-enrichment is the ratio of these proportions and represents how much more/less of the CNVs

overlap an exon compared to the control regions. The boxplot in Figure 2c shows the distribution of the 100 sub-sampling in each cohort.

To test if the difference observed between the cohort is significant, the *cohort* labels were permuted 10,000 times and the difference in median across the 100 sub-sampling was saved. The resulting P-value was computed as $\frac{1+d}{1+N}$ where d is the number of times the permuted difference was greater or equal to the observed difference, and N is the number of permutations.

The same analysis was repeated for exons from genes with a probability of loss-of-function intolerance[17] higher than 0.9. These genes were called *LoF intolerant genes* in Figure 2c. Small ($<$ 50Kbp) and large ($>$50 Kbp) CNVs were analyzed separately. The analysis was repeated using rare CNVs only.

Selecting control regions The control regions must have the same size distribution as the regions they are derived from (e.g. CNVs in a CNV catalog). We also controlled for the overlap with centromere, telomeres and assembly gaps (CTGs) to avoid selecting control regions in assembly gaps where no CNV or annotation is available. To select control regions, thousands of bases were first randomly chosen in the genome. The distance between each base and the nearest CTG was then computed. At this point, selecting a region of a specific size and with specific overlap profile can be done by randomly choosing as center one of the bases that fit the profile:

$$\left\{b, O_{CTG}(d^b_{CTG} - \frac{S_r}{2}) < 0\right\} \tag{1}$$

with O_{CTG} equals 1 if the original region overlaps with a CTG, -1 if not; d^b_{CTG} is the distance between base b and the nearest CTG; and S_r is the size of the original region. For each input region, a control region was selected and had by construction the exact same size and overlap profile.

Recurrence of rare exonic CNVs In each cohort, we retrieved the CNV catalog of rare ($<$1% in all 5 public datasets) exonic CNVs. We annotated each CNV with its recurrence in the cohort. We then evaluated the proportion of the CNVs in the catalog that are private (i.e. seen in only one sample), or seen in X samples or more. This cumulative proportion of CNVs is shown in S12 Fig.

The control cohort was down-sampled a thousand times to the same sample size as the epilepsy cohort. These down-sampling provided a confidence interval (ribbon in S12 Fig) and an empirical P-value.

We performed the same analysis after removing the top 20 samples with the most non-private rare exonic CNVs (S12 Fig). With this analysis, we tried to remove the potential effect of a few extreme samples.

We also repeated the analysis using only French-Canadians individuals, to ensure that the observed differences are not caused by rare population-specific variants (S12 Fig).

CNVs and epilepsy genes We used the list of genes associated with epilepsy from the EpilepsyGene resource[18] which consists of 154 genes strongly associated with epilepsy. For a particular set of CNV we count how many of the genes hit are known epilepsy genes. We noticed that the epilepsy genes tend to be large, and genes hit by CNVs also (S13 Fig). This could lead to a spurious association so we also performed a permutation approach that controls for the size of the genes. To control for the gene size of epilepsy genes and CNV-hit genes, we randomly selected genes with sizes similar to the genes hit by CNVs and evaluated how many of these were epilepsy genes. After ten thousand samplings, we computed an empirical P-value. The permutation P-value was computed as $\frac{1+d}{1+N}$ where d is the number of times the number of epilepsy genes in the random set of genes was greater or equal to the one in genes hit by CNVs, and N is the number of permutations. Using this sampling approach we tested different sets of CNVs: deletion or duplications of different frequencies in the epilepsy cohort, control individuals and samples from the twin study.

To investigate rare non-coding CNV close to known epilepsy genes, we counted how many patients have such a CNV at different distance thresholds. For example, how many patients had a rare non-coding CNV at 10 Kbp of an epilepsy gene's exon or closer. We compared this cumulative distribution to the control cohort, after down-sampling it to the sample size of the epilepsy cohort. Down-sampling was also used to produce a confidence interval, represented by the ribbon in Figure 3c. This analysis was repeated using deletions only. Each epilepsy gene was also tested for an excess of rare non-coding deletions in patients versus controls using a Fisher test.

In order to retrieve non-coding CNV that might have a functional impact, we downloaded eQTLs

associated with the epilepsy genes, as well as DNase 1 hypersensitive sites associated with the promoter of epilepsy genes. The eQTLs are provided by the GTEx project[19]. Pairs of associated DNase 1 hypersensitive sites and associated genes[20] were downloaded at http://www.uwencode.org/proj/Science_Maurano_Humbert_et_al/data/genomewideCorrs_above0.7_promoterPlusMinus500kb_withGeneNames_35celltypeCategories.bed8.gz.

A Kolmogorov-Smirnov test was used to compare the distance distributions in epilepsy patients versus controls. We also computed the odds ratio of having such a CNV for different distance thresholds between epilepsy patients and controls. For a distance d, we computed:

$$OR = \frac{S^{CNV}_{patient}}{S^{CNV}_{control}} / \frac{S^{noCNV}_{patient}}{S^{noCNV}_{control}}$$

where $S^{CNV}_{patient}$ is the number of patients with a rare non-coding CNV overlapping a functional region and located at d bp or less from the exon of a known epilepsy gene.

References

[1] Scheinin I, Sie D, Bengtsson H, van de Wiel MA, Olshen AB, van Thuijl HF, et al. DNA copy number analysis of fresh and formalin-fixed specimens by shallow whole-genome sequencing with identification and exclusion of problematic regions in the genome assembly. Genome research. 2014;24(12):2022–32. doi:10.1101/gr.175141.114.

[2] Redon R, Ishikawa S, Fitch KR, Feuk L, Perry GH, Andrews TD, et al. Global variation in copy number in the human genome. Nature. 2006;444(7118):444–454. doi:10.1038/nature05329.

[3] Itsara A, Cooper GM, Baker C, Girirajan S, Li J, Absher D, et al. Population Analysis of Large Copy Number Variants and Hotspots of Human Genetic Disease. The American Journal of Human Genetics. 2009;84(2):148–161. doi:10.1016/j.ajhg.2008.12.014.

[4] Addis L, Rosch RE, Valentin A, Makoff A, Robinson R, Everett KV, et al. Analysis of rare copy number variation in absence epilepsies. Neurology Genetics. 2016;2(2):e56. doi:10.1212/NXG.0000000000000056.

[5] Conrad DF, Pinto D, Redon R, Feuk L, Gokcumen O, Zhang Y, et al. Origins and functional impact of copy number variation in the human genome. Nature. 2010;464(7289):704–712. doi:10.1038/nature08516.

[6] Li H, Durbin R. Fast and accurate long-read alignment with Burrows-Wheeler transform. Bioinformatics. 2010;26(5):589–595. doi:10.1093/bioinformatics/btp698.

[7] Seshan V, Olshen A. DNAcopy: DNA copy number data analysis. R package version 1501. 2017;.

[8] Boeva V, Zinovyev A, Bleakley K, Vert JP, Janoueix-Lerosey I, Delattre O, et al. Control-free calling of copy number alterations in deep-sequencing data using GC-content normalization. Bioinformatics. 2011;27(2):268–269. doi:10.1093/bioinformatics/btq635.

[9] Abyzov A, Urban AE, Snyder M, Gerstein M. CNVnator: An approach to discover, genotype, and characterize typical and atypical CNVs from family and population genome sequencing. Genome Research. 2011;21(6):974–984. doi:10.1101/gr.114876.110.

[10] Klambauer G, Schwarzbauer K, Mayr A, Clevert DA, Mitterecker A, Bodenhofer U, et al. Cn.MOPS: Mixture of Poissons for discovering copy number variations in next-generation sequencing data with a low false discovery rate. Nucleic Acids Research. 2012;40(9):e69–e69. doi:10.1093/nar/gks003.

[11] Layer RM, Chiang C, Quinlan AR, Hall IM. LUMPY: a probabilistic framework for structural variant discovery. Genome Biology. 2014;15(6):R84. doi:10.1186/gb-2014-15-6-r84.

[12] Faust GG, Hall IM. YAHA: Fast and flexible long-read alignment with optimal breakpoint detection. Bioinformatics. 2012;28(19):2417–2424. doi:10.1093/bioinformatics/bts456.

[13] Handsaker RE, Van Doren V, Berman JR, Genovese G, Kashin S, Boettger LM, et al. Large multiallelic copy number variations in humans. Nature Genetics. 2015;47(3):296–303. doi:10.1038/ng.3200.

[14] Sudmant PH, Rausch T, Gardner EJ, Handsaker RE, Abyzov A, Huddleston J, et al. An integrated map of structural variation in 2,504 human genomes. Nature. 2015;526(7571):75–81. doi:10.1038/nature15394.

[15] Francioli LC, Menelaou A, Pulit SL, van Dijk F, Palamara PF, Elbers CC, et al. Whole-genome sequence variation, population structure and demographic history of the Dutch population. Nature Genetics. 2014;46(8):818–825. doi:10.1038/ng.3021.

[16] Sudmant PH, Mallick S, Nelson BJ, Hormozdiari F, Krumm N, Huddleston J, et al. Global diversity, population stratification, and selection of human copy-number variation. Science. 2015;349(6253):aab3761–aab3761. doi:10.1126/science.aab3761.

[17] Lek M, Karczewski KJ, Minikel EV, Samocha KE, Banks E, Fennell T, et al. Analysis of protein-coding genetic variation in 60,706 humans. Nature. 2016;536(7616):285–291. doi:10.1038/nature19057.

[18] Ran X, Li J, Shao Q, Chen H, Lin Z, Sun ZS, et al. EpilepsyGene: A genetic resource for genes and mutations related to epilepsy. Nucleic Acids Research. 2015;43(D1):D893–D899. doi:10.1093/nar/gku943.

[19] Ardlie KG, Deluca DS, Segre AV, Sullivan TJ, Young TR, Gelfand ET, et al. The Genotype-Tissue Expression (GTEx) pilot analysis: Multitissue gene regulation in humans. Science. 2015;348(6235):648–660. doi:10.1126/science.1262110.

[20] Maurano MT, Humbert R, Rynes E, Thurman RE, Haugen E, Wang H, et al. Systematic Localization of Common Disease-Associated Variation in Regulatory DNA. Science. 2012;337(6099):1190–1195. doi:10.1126/science.1222794.
