# Supplementary material for: Global characterization of copy number variants in epilepsy patients from whole genome sequencing
Source: PLoS Genet. 2018 Apr 12;14(4):e1007285. doi: 10.1371/journal.pgen.1007285 (PMC5978987; doi:10.1371/journal.pgen.1007285)
Supplement: S3 Table — (PDF) [file pgen.1007285.s004.pdf]

Patient	Epilepsy type	Syndrome	Copy number	Chr.	CNV start	CNV end	Exon disrupted	Taqman probe
CNET0188	Focal	Mesial temporal lobe sclerosis	1	2	141335001	141365000	*LRP1B*	Hs02078420_cn
CNET0084	Focal	Temporal lobe	1	4	120205001	120280000	*USP53;FABP2;C4orf3*	Hs04813260_cn
CNET0143	Generalized	Childhood absence epilepsy	1	5	65055001	65465000	*NLN;ERBB2IP;SREK1*	Hs03552554_cn
CNET0151	Generalized	Eyelid myoclonia epilepsy with absence	1	9	8600001	8770000	*PTPRD*	Hs06875003_cn
CNET0041	Generalized	Idiopathic generalized epilepsies	1	11	62625001	62645000	*SLC3A2*	Hs03777991_cn
CNET0066	Generalized	Idiopathic generalized epilepsies	1	13	67325001	67575000	*PCDH9*	Hs06378870_cn
CNET0025	Generalized	Early onset absence epilepsy (onset <4, absence with or without GTCs)	1	15	60735001	60805000	*RORA;NARG2*	Hs05369880_cn
CNET0195	Focal	Occipital lobe epilepsy	1	22	34095001	34200000	*LARGE*	Hs05575584_cn
CNET0005	Generalized	febrile sz, child onset GTCs	1	22	41960001	42050000	*PMM1;DESI1;CSDC2;XRCC6*	Hs05580065_cn
