# Supplementary figures and images for: Global characterization of copy number variants in epilepsy patients from whole genome sequencing
Source: PLoS Genet. 2018 Apr 12;14(4):e1007285. doi: 10.1371/journal.pgen.1007285 (PMC5978987; doi:10.1371/journal.pgen.1007285)

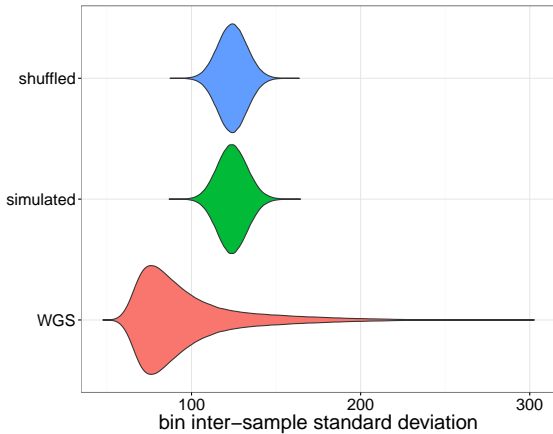

(a)

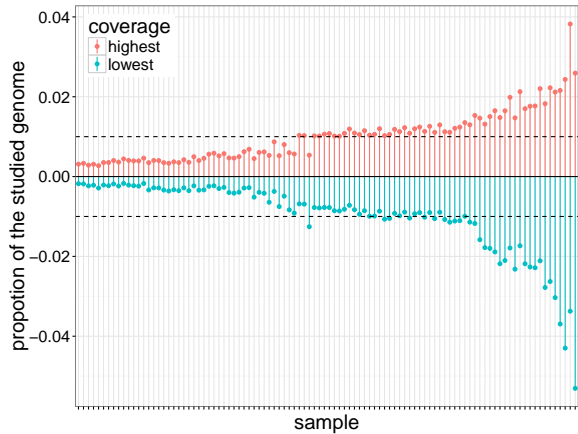

(b)

Supplement: S1 Fig — a) Distribution of the bin inter-sample standard deviation coverage (red) and null distribution (blue: bins shuffled, green: simulated normal distribution). b) Proportion of the genome in which a given sample (x-axis) has the highest (red) or lowest (blue) RD. In the absence of bias all samples should be the most extreme at the same frequency (dotted horizontal line). (PDF) [file pgen.1007285.s005.pdf]

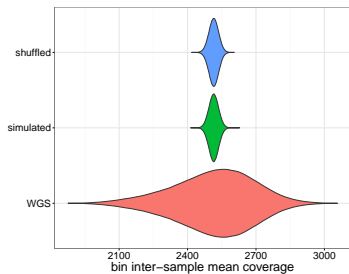

(a)

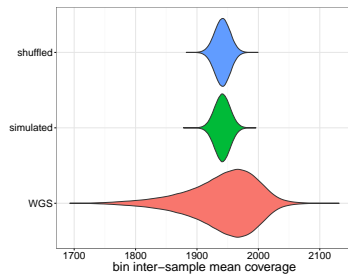

(b)

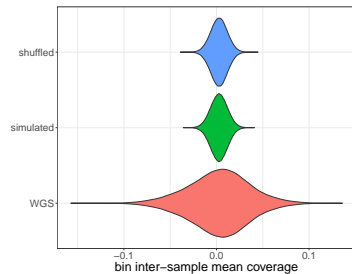

(c)

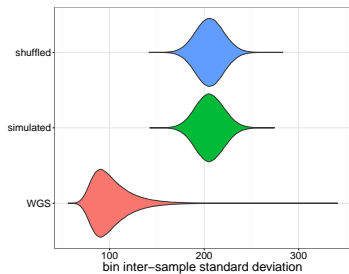

(d)

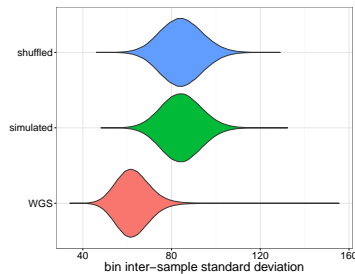

(e)

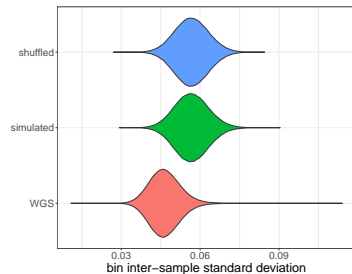

(f)

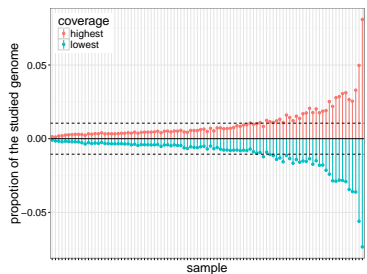

(g)

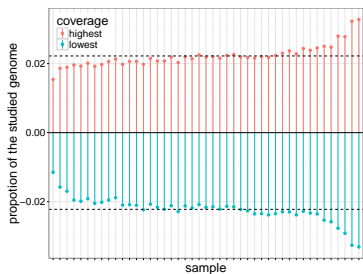

(h)

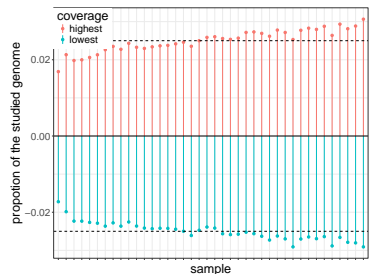

(i)

Supplement: S2 Fig — a-c) Distribution of the bin inter-sample standard deviation coverage (red) and null distribution (blue: bins shuffled, green: simulated normal distribution). d-f) Same for the bin inter-sample standard deviation coverage. g-i) Proportion of the genome in which a given sample (x-axis) has the highest (red) or lowest (blue) RD. In the absence of bias all samples should be the most extreme at the same frequency (dotted horizontal line). (PDF) [file pgen.1007285.s006.pdf]

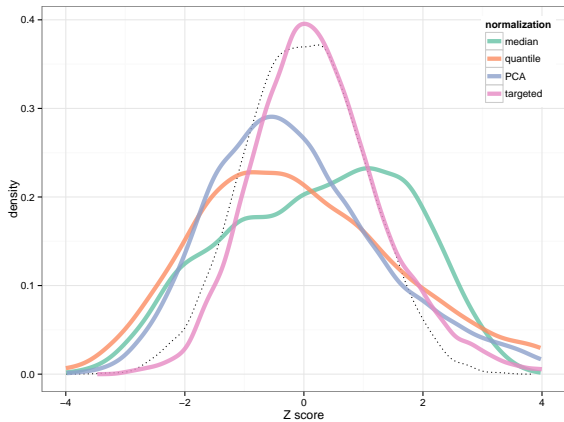

(a)

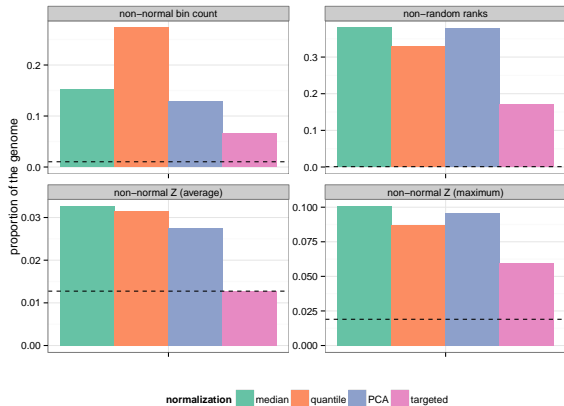

(b)

Supplement: S3 Fig — a) For each normalization approach, the sample with the least normal Z-score distribution is shown. b) After targeted normalization, a lower proportion of the genome looks problematic for the analysis. Fewer bins have non-normal bin counts (top-left), the sample ranks are more random suggesting less sample-specific bias (top-right), and Z-scores fit better a Normal distribution on average (bottom-left) and in the worst sample (bottom-right). The dotted line is computed from simulated bin counts. (PDF) [file pgen.1007285.s007.pdf]

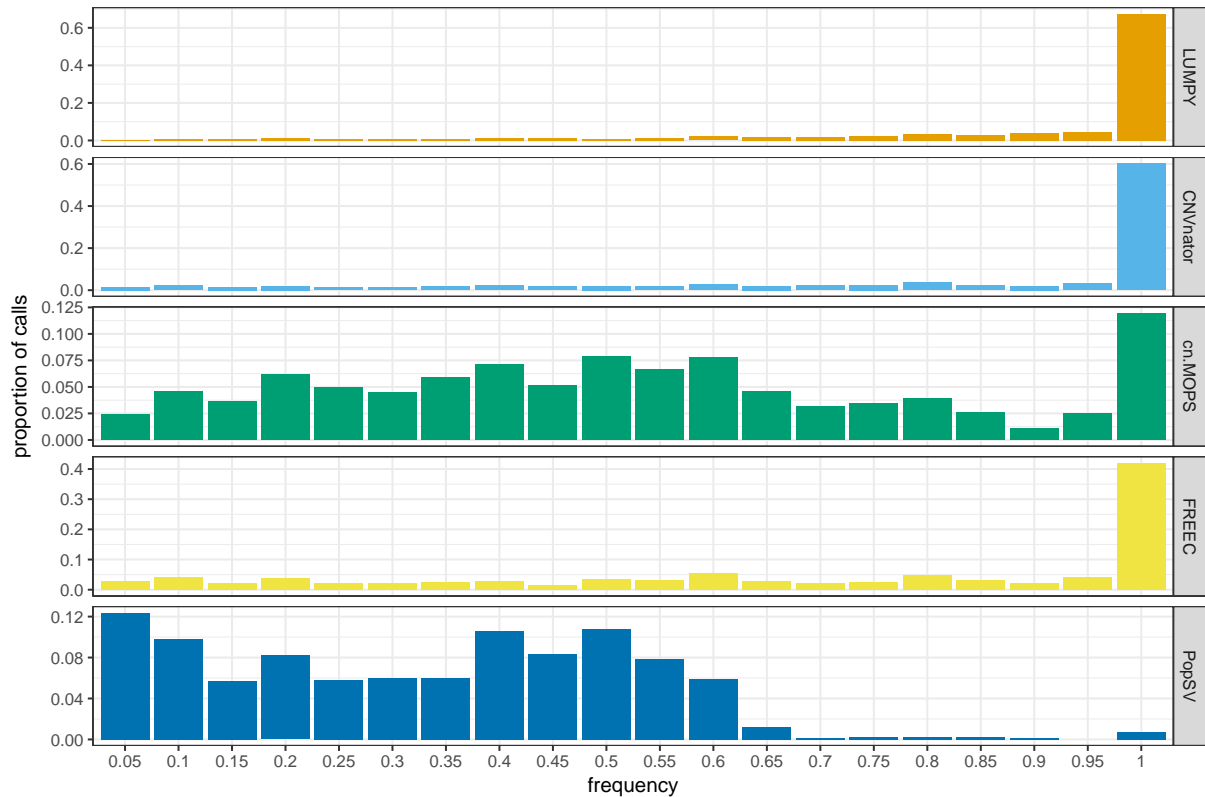

Supplement: S4 Fig — The bars show the proportion of calls in an average samples (y-axis), grouped by the frequency of the call in the dataset (x-axis), for different methods. (PDF) [file pgen.1007285.s008.pdf]

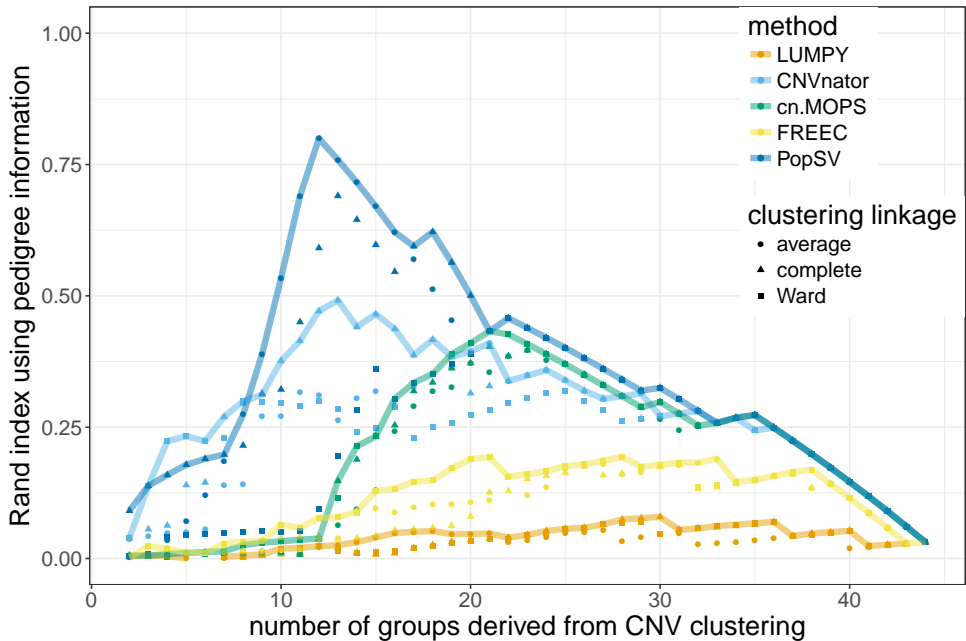

Supplement: S5 Fig — The hierarchical cluster tree from the CNV calls is cut at different levels (x-axis), cluster groups are compared to the known pedigree using the Rand index (y-axis). Different clustering linkage criteria (point style) are used and the one showing the best Rand index is highlighted by the line. (PDF) [file pgen.1007285.s009.pdf]

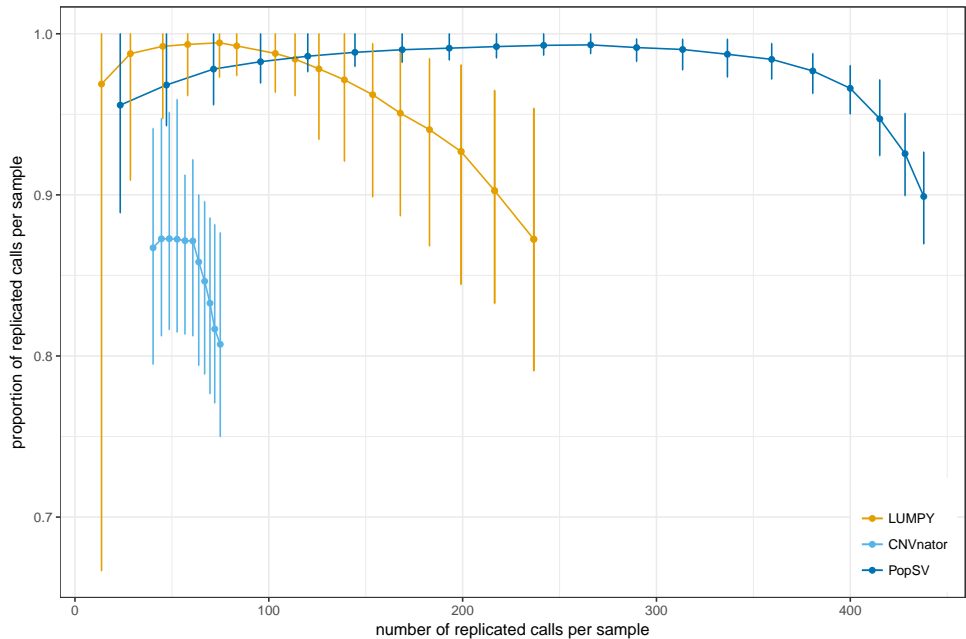

Supplement: S6 Fig — Each point represents the number of replicated calls per sample (average across samples) and the proportion of replicated calls per sample. The vertical error bar shows the variation of the replication rate across the samples. The points and lines were computed by filtering calls at different significance levels (q-value for PopSV, number of supporting reads for LUMPY and eval1/eval2 for CNVnator, see S1 Text). (PDF) [file pgen.1007285.s010.pdf]

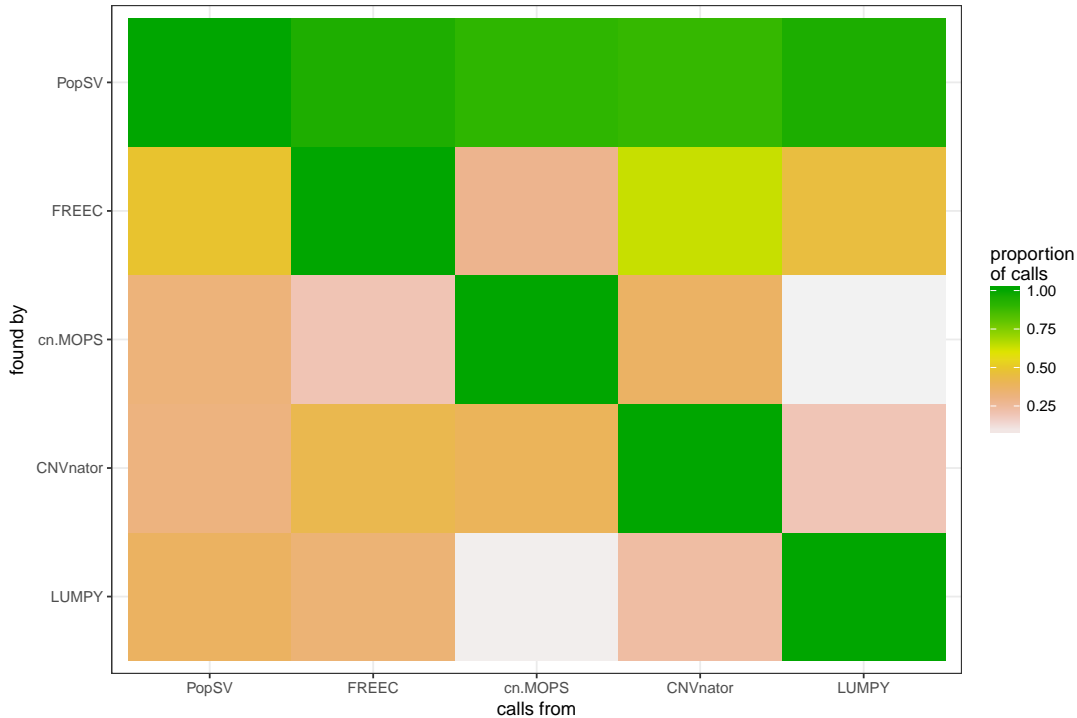

Supplement: S7 Fig — Focusing on calls found by at least two methods, the heatmap shows the proportion of calls from one method (x-axis) that were also found by another (y-axis) on average per sample. (PDF) [file pgen.1007285.s011.pdf]

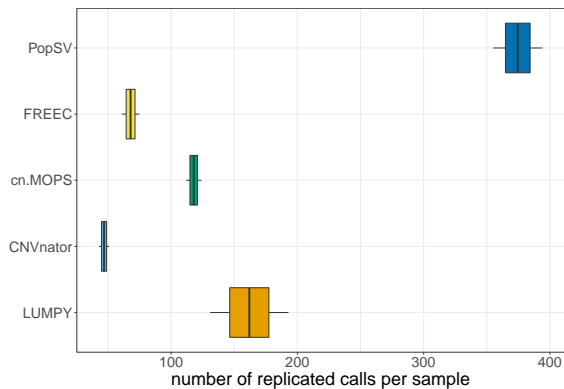

(a)

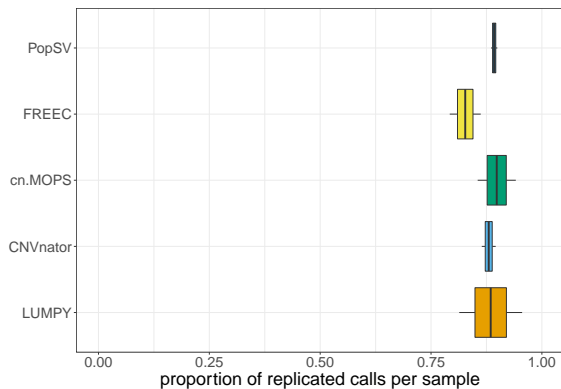

(b)

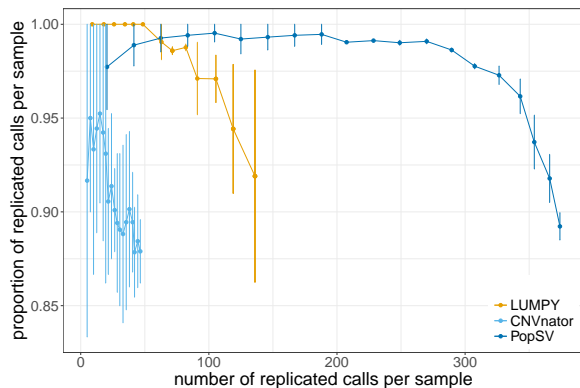

(c)

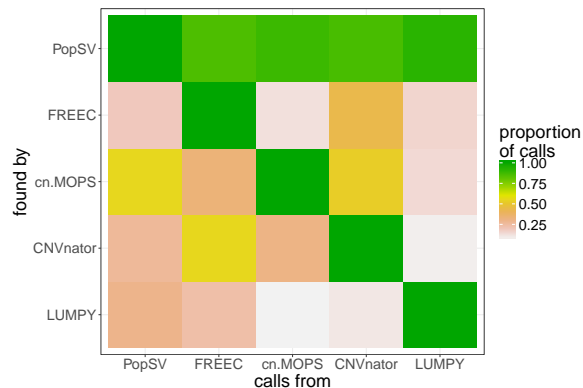

(d)

Supplement: S8 Fig — Number (a) and proportion (b) of germline calls replicated in the paired tumor in CageKid. c) Number and proportion of replicated calls when filtering calls at different significance levels. d) Focusing on calls found by at least two methods, the color shows the proportion of calls from one method (x-axis) that were also found by another (y-axis) on average per sample. (PDF) [file pgen.1007285.s012.pdf]

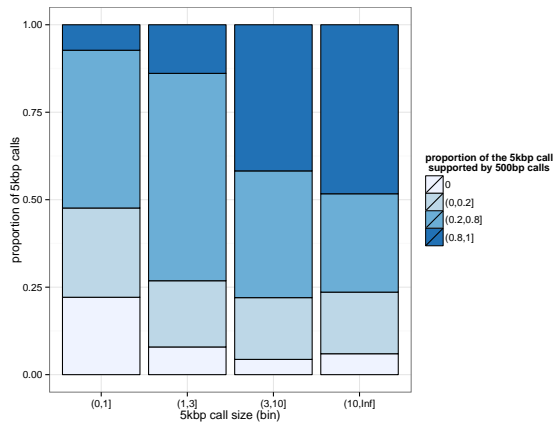

(a)

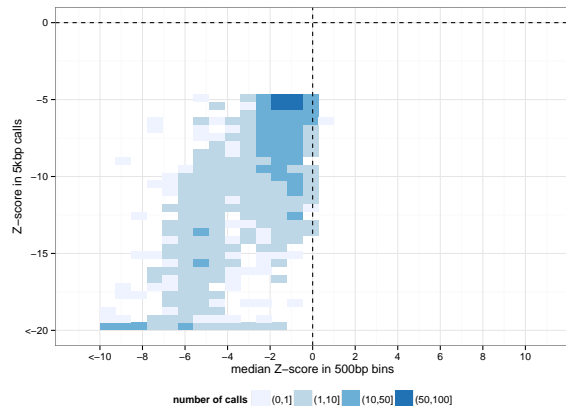

(b)

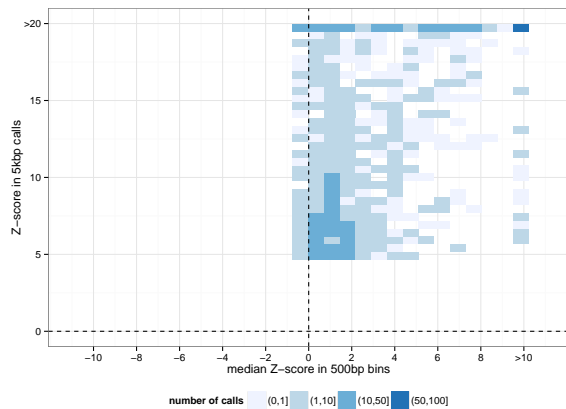

(c)

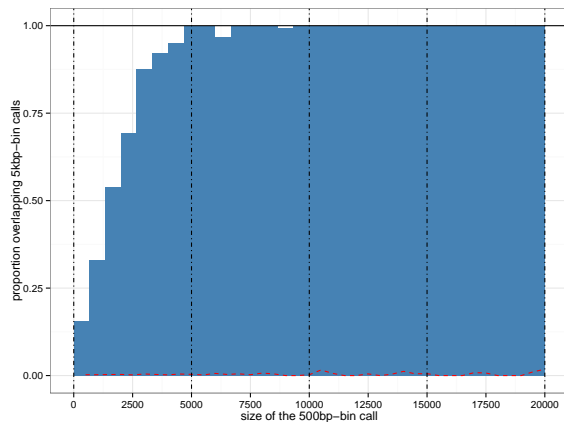

(d)

Supplement: S9 Fig — a) 5 Kbp calls of different sizes (x-axis) are split according to the proportion of the call supported by 500 bp calls. The Z-score of 500 bp bins in 5 Kbp calls is consistent with the call for deletion b) and duplication c) signal. 5 Kbp calls with lower significance (e.g. single-bin calls) are less supported by 500 bp calls (a) but their Z-scores are in the consistent direction (b,c) although not always significant enough to be called. d) Proportion of 500 bp calls of different sizes (x-axis) overlapping a 5 Kbp call. (PDF) [file pgen.1007285.s013.pdf]

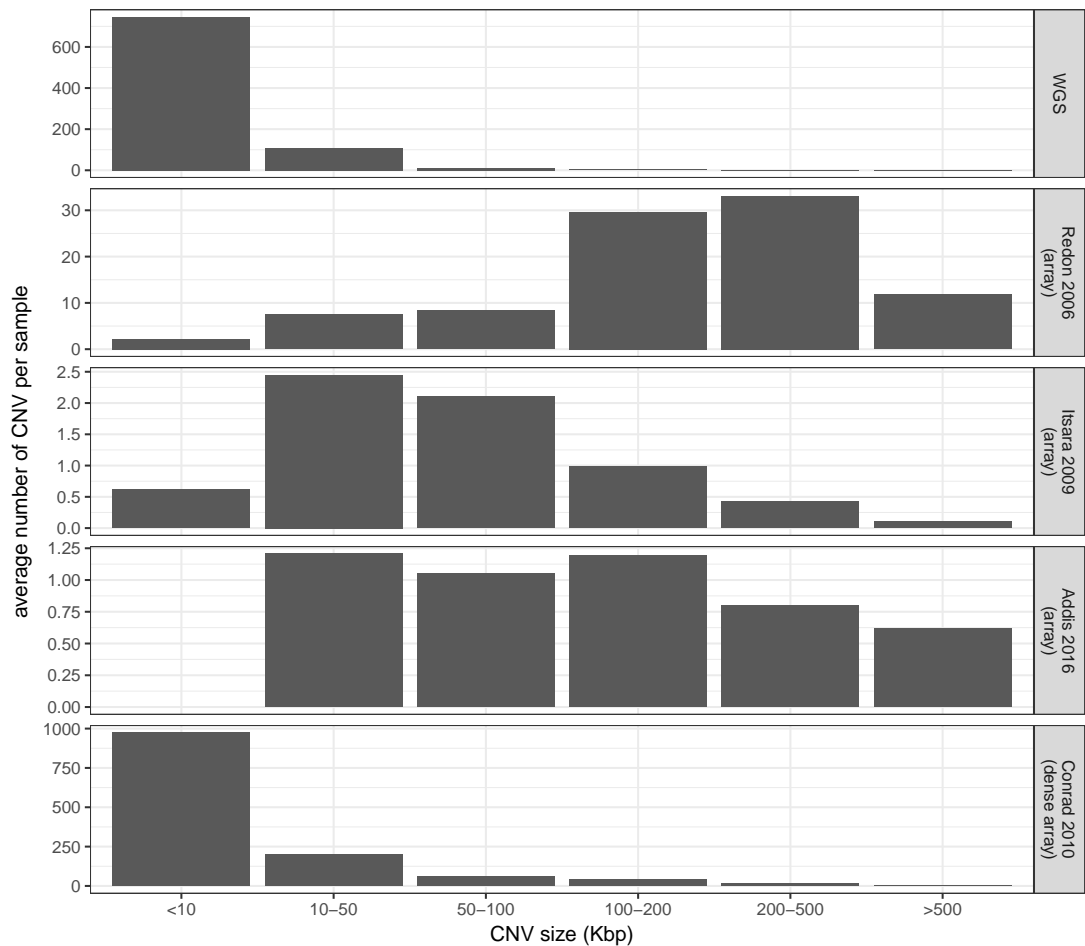

Supplement: S10 Fig — The bars show the average number of CNVs called in a sample in the different cohorts. Redon 2006 [42] and Itsara 2009 [43] are population studies using technology similar to previous epilepsy studies. Addis 2016 [34] is a recent study of large CNVs in absence epilepsy. Conrad 2010 [4] is a population study that used multiple arrays to increase its resolution. (PDF) [file pgen.1007285.s014.pdf]

difference between patients and controls

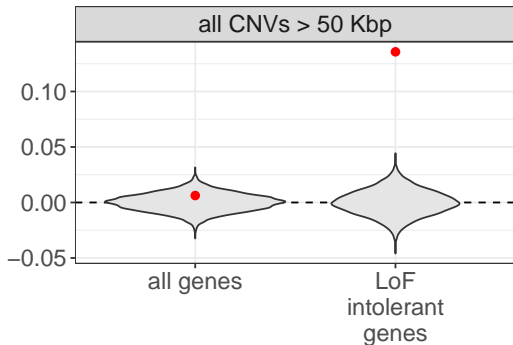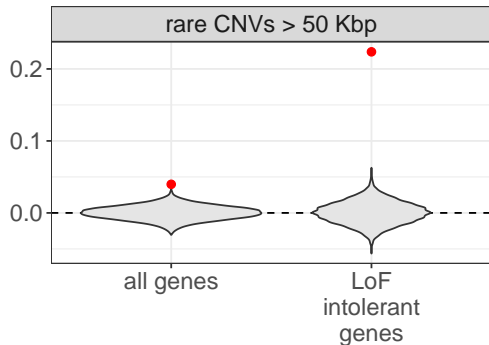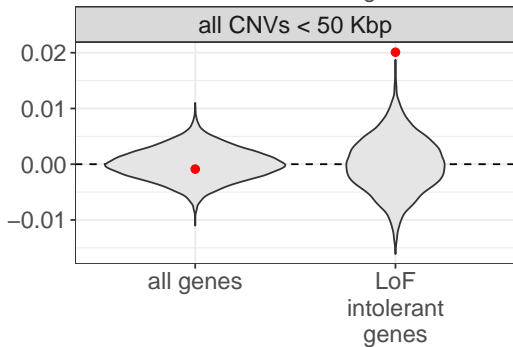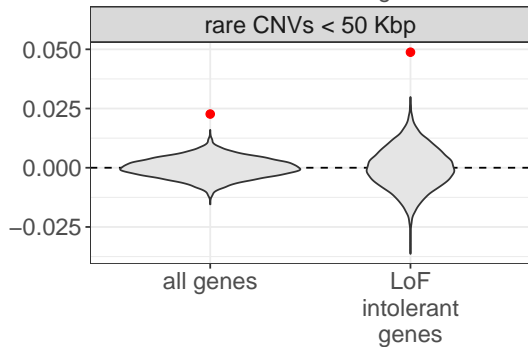

Supplement: S11 Fig — The grey violin plot represents the difference in fold-enrichment between patients and controls across 10,000 permutations where the patient/control labels had been shuffled. The red point represents the observed difference between patients and controls (Fig 2c). (PDF) [file pgen.1007285.s015.pdf]

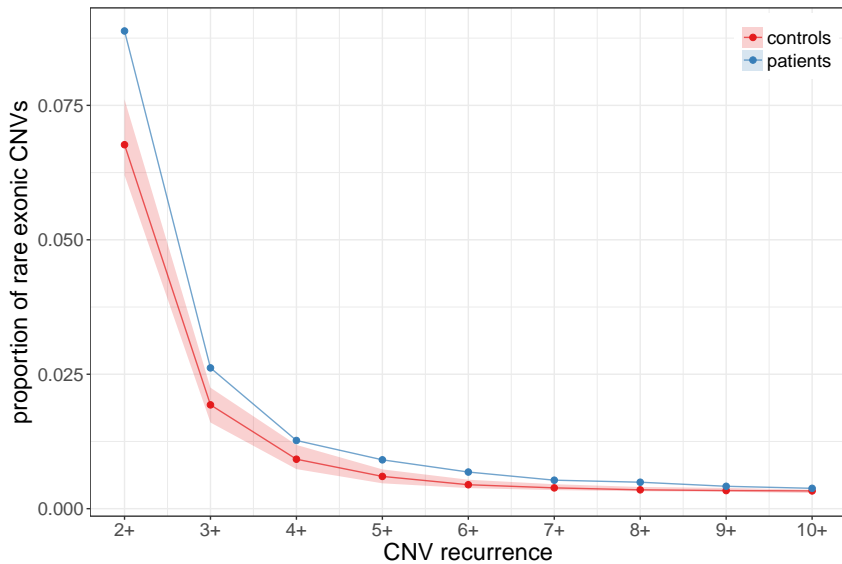

(a)

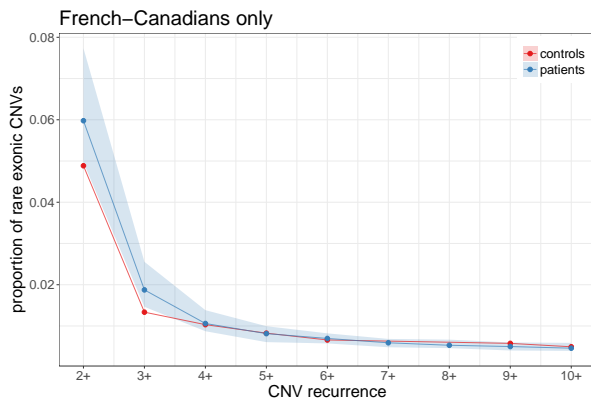

(b)

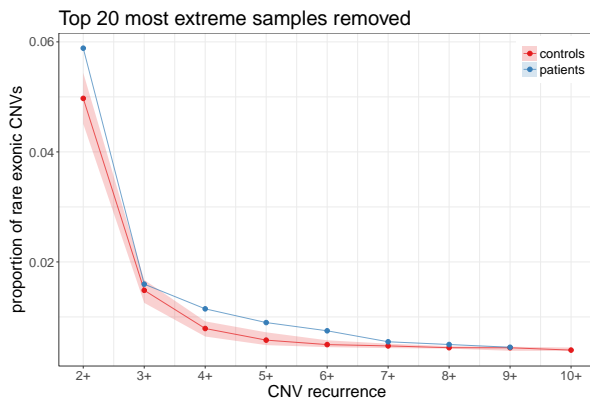

(c)

Supplement: S12 Fig — Proportion of rare exonic CNVs (y-axis) seen in X or more individuals (x-axis). The ribbon shows the 5%–95% confidence interval. In b), only French-Canadians individuals were analyzed and we down-sampled the epilepsy cohort to match the sample size of the French-Canadians controls. In c), the top 20 samples with the most non-private rare exonic SVs were removed. (PDF) [file pgen.1007285.s016.pdf]

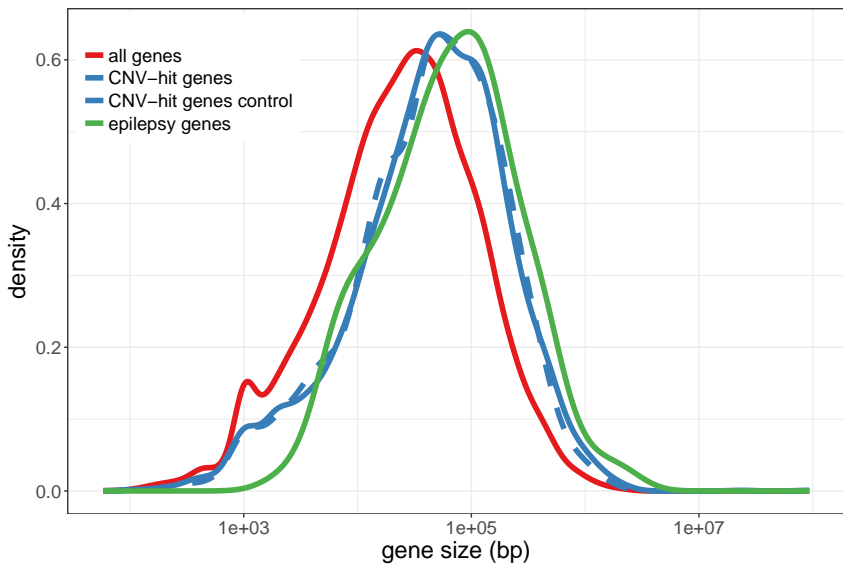

(a)

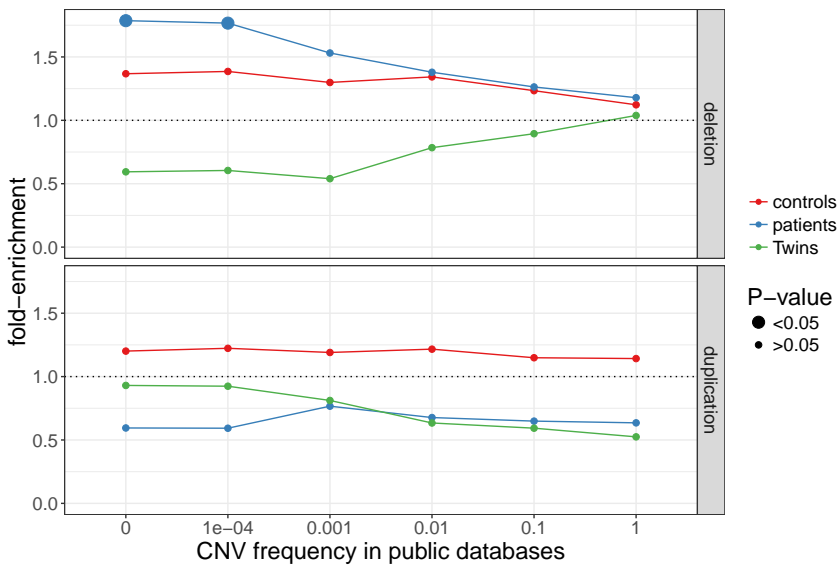

(b)

Supplement: S13 Fig — a) Epilepsy genes (red) are genes known to be associated with epilepsy. The control genes (dotted blue) are random genes selected so that the size distribution is similar to the sizes of genes hit by CNVs (plain blue). b) In three different datasets (color), genes hit by rare deletion (top) or duplications (bottom) at different frequency thresholds (x-axis) were tested for enrichment in epilepsy genes (y-axis, point-size). (PDF) [file pgen.1007285.s017.pdf]

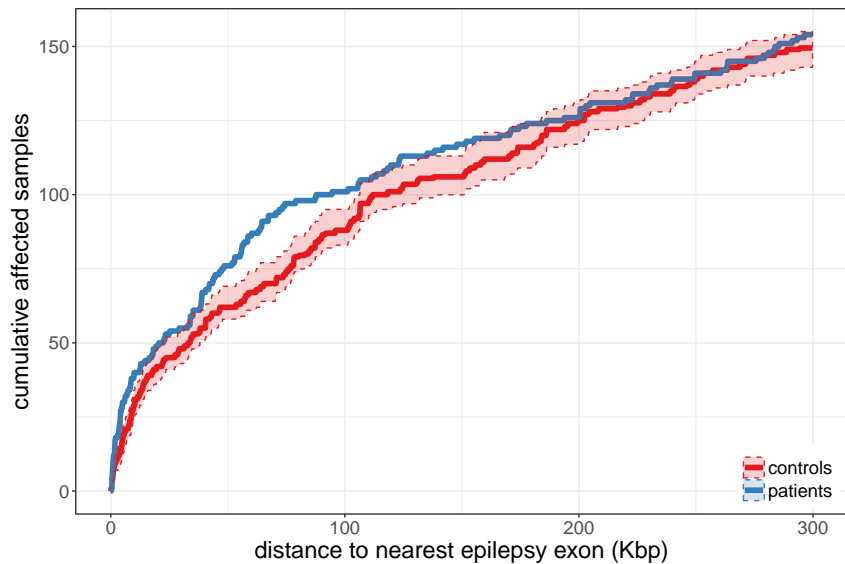

(a) Rare CNVs

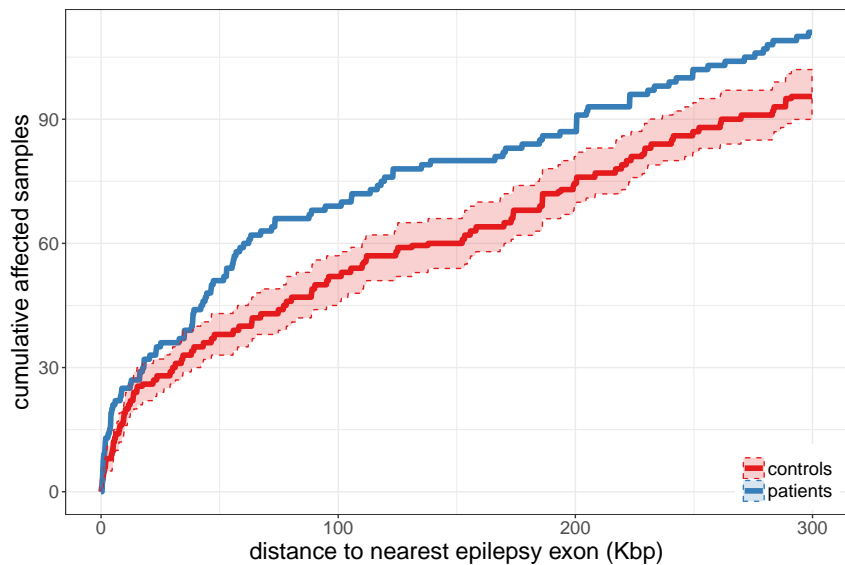

(b) Rare deletions

Supplement: S14 Fig — The graphs show the cumulative number of individuals (y-axis) with a rare non-coding variants located at X Kbp or less (x-axis) from the exonic sequence of a known epilepsy gene. The controls were down-sampled to the sample size of the epilepsy cohort. The ribbon shows the 5%/95% confidence interval. In a), deletions and duplications were considered; in b), only deletions were used. (PDF) [file pgen.1007285.s018.pdf]

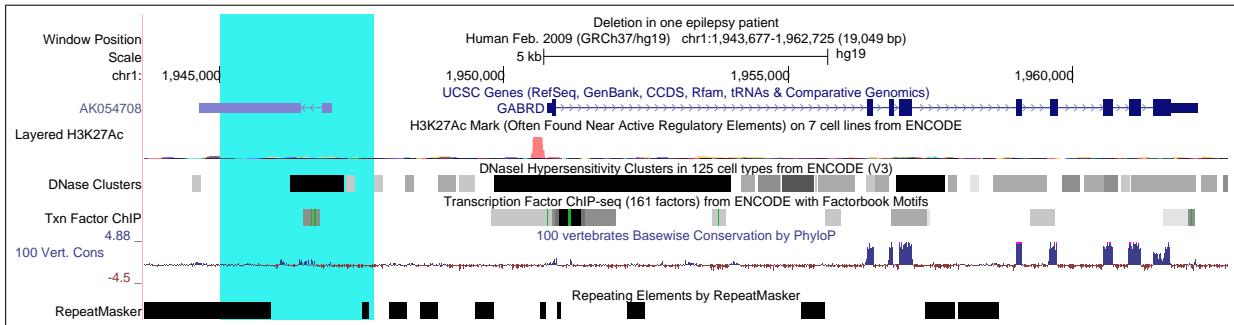

(a)

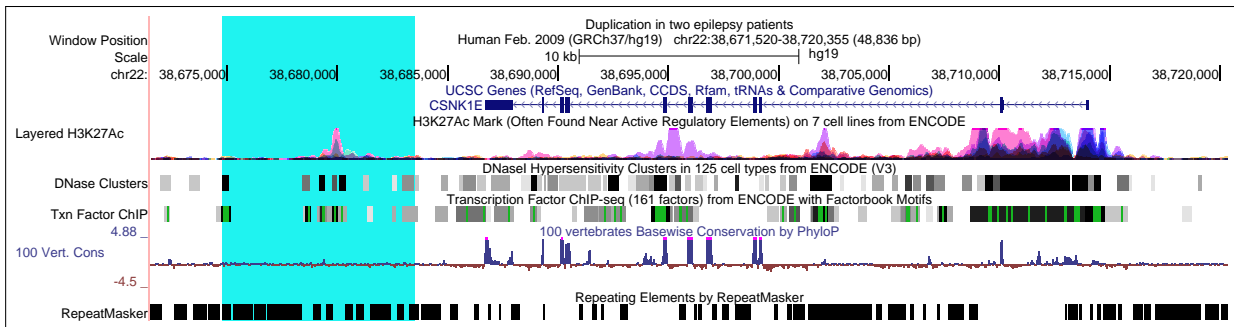

(b)

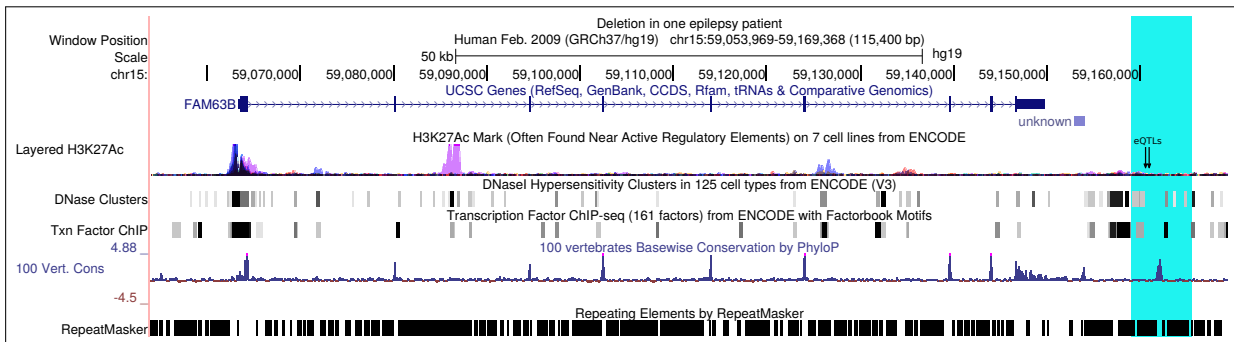

(c)

Supplement: S15 Fig — a) 2.7 Kbp deletion in an epilepsy patient, never seen in controls or CNV databases. Three other epilepsy patients have a rare non-coding deletions located at less than 200 Kbp from the GABRD gene. b) 8.8 Kbp duplication in two epilepsy patients, never seen in controls or CNV databases and overlapping a regulatory region associated with CSNK1E. c) 6.5 Kbp deletion of an ultra-conserved regions downstream of FAM63B. Two expression QTLs for this gene are highlighted with arrows. (PDF) [file pgen.1007285.s019.pdf]

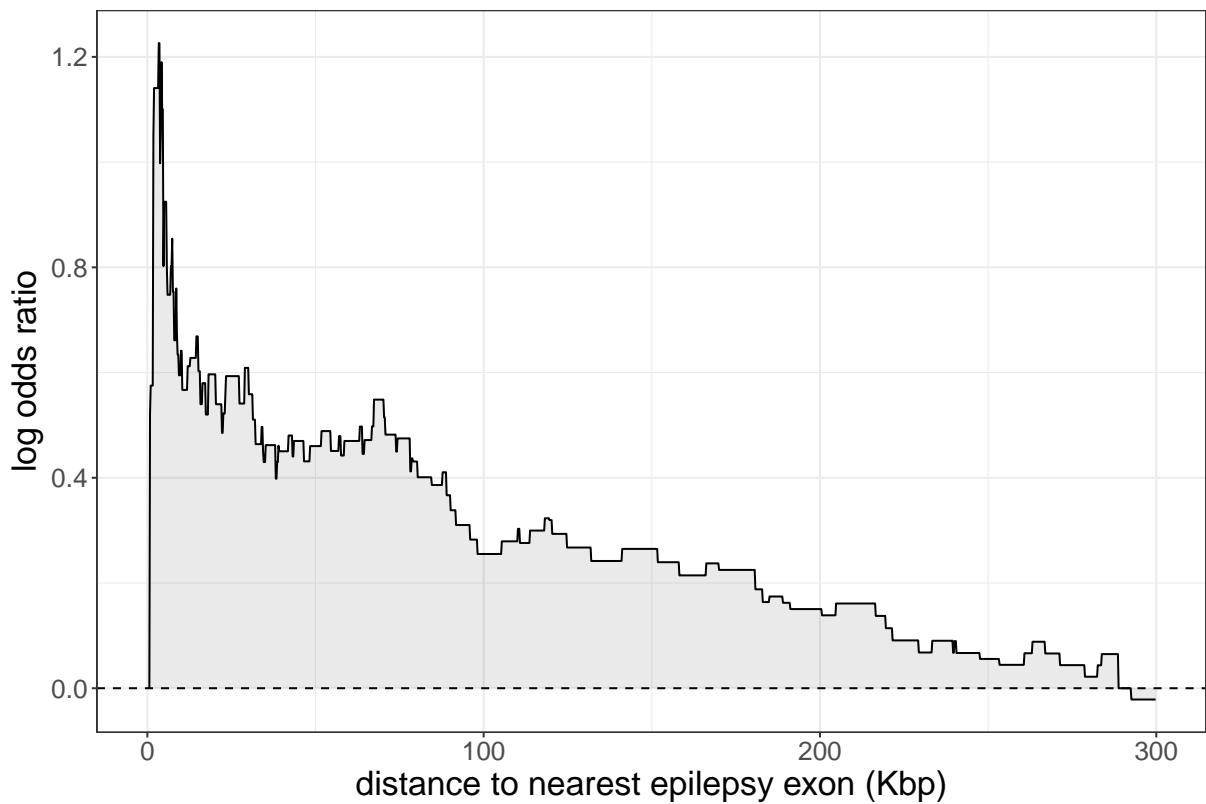

Supplement: S16 Fig — The graph shows the log odds ratio of having a rare non-coding CNV located at X Kbp or less (x-axis) from the exonic sequence of a known epilepsy gene. The y-axis shows the log odds ratio between epilepsy patients and controls. The controls were down-sampled to the sample size of the epilepsy cohort. We used CNVs overlapping regions functionally associated with the epilepsy gene (eQTL or promoter-associated DNase site). (PDF) [file pgen.1007285.s020.pdf]

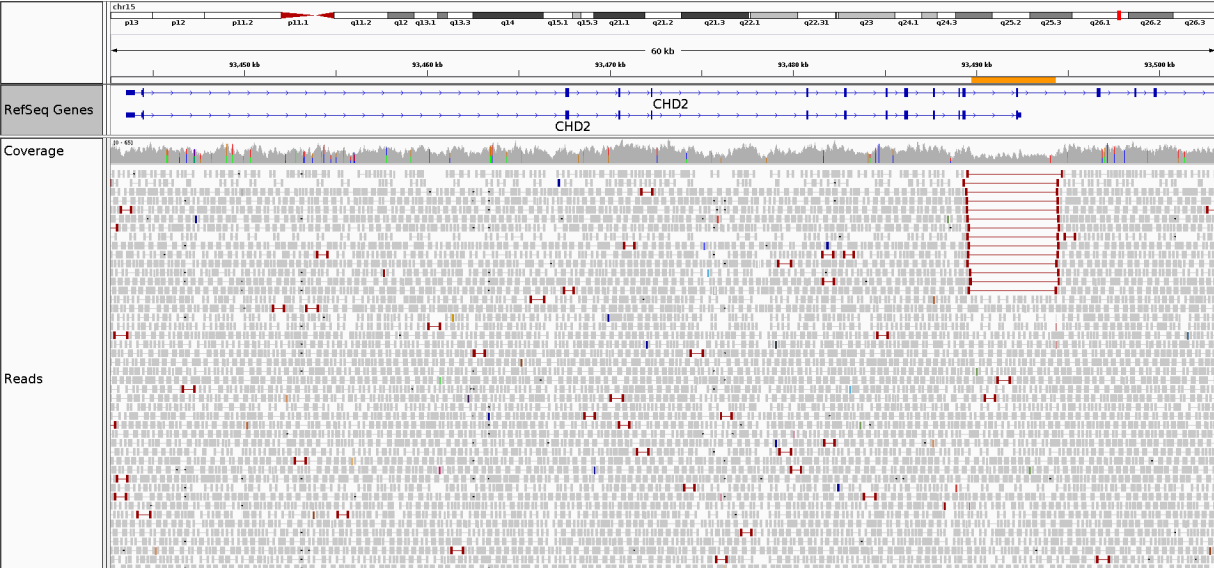

Supplement: S17 Fig — Abnormal mapping of the read pairs highlighted in red support the deletion detected by PopSV using the read coverage. The deletion region is highlighted in orange. (PDF) [file pgen.1007285.s021.pdf]

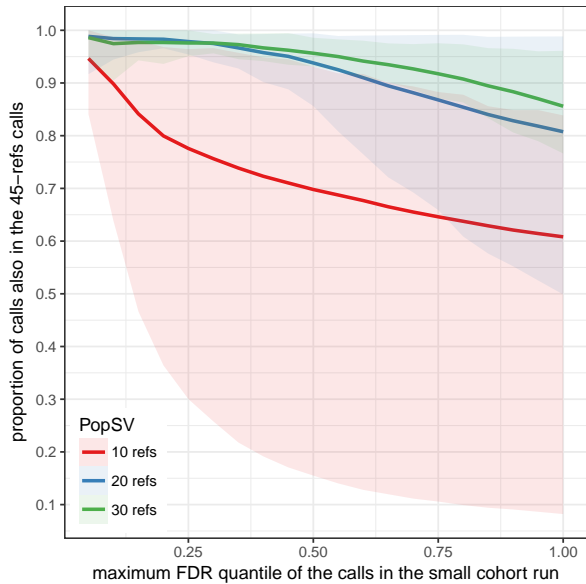

(a)

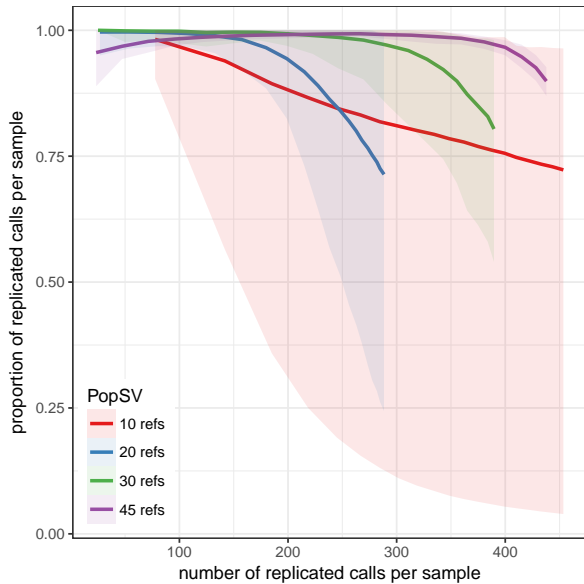

(b)

Supplement: S18 Fig — PopSV was run on the Twins study using 10, 20, 30 or 45 samples as reference (color). In a), the y-axis shows how many calls from the down-sampled run were found in the original 45-refs run. The x-axis represents the FDR threshold (lower threshold being more stringent). b) Replication in monozygotic twins. For different cohort sizes and FDR thresholds, the number (x-axis) and proportion (y-axis) of calls replicated in the other monozygotic twin is shown. In both graphs, the lines represents the median per sample and the ribbon the minimum/maximum values. (PDF) [file pgen.1007285.s022.pdf]

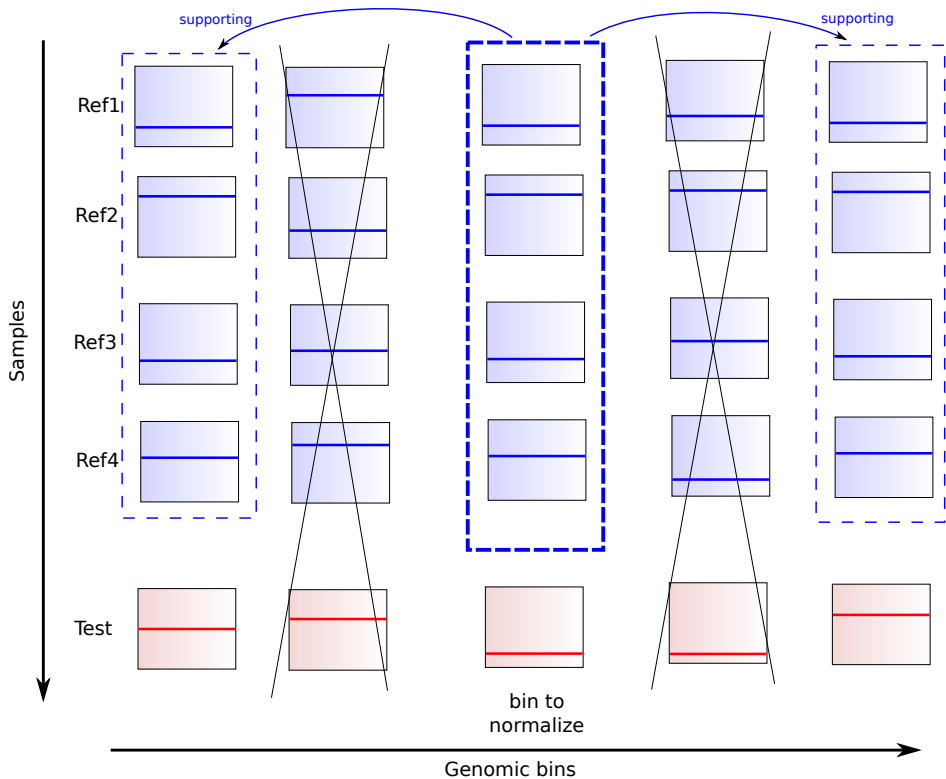

Supplement: S19 Fig — The coverage across the reference samples (blue) in the bin to normalize is used to find supporting bins across the genome. These supporting bins only are used to compute the normalization factor. The same supporting bins will be used to normalize the bin count in a test sample (red). (PDF) [file pgen.1007285.s023.pdf]
